# LSD1 inhibition attenuates androgen receptor V7 splice variant activation in castration resistant prostate cancer models

**DOI:** 10.1186/s12935-018-0568-1

**Published:** 2018-05-09

**Authors:** Sergio Regufe da Mota, Sarah Bailey, Rosemary A. Strivens, Annette L. Hayden, Leon R. Douglas, Patrick J. Duriez, M. Teresa Borrello, Hanae Benelkebir, A. Ganesan, Graham Packham, Simon J. Crabb

**Affiliations:** 1 Cancer Sciences Unit and Cancer Research UK Centre, University of Southampton, Southampton General Hospital, Somers Cancer Research Building, Mailpoint 824, Southampton, SO16 6YD UK; 2 Protein Core Facility, Cancer Research UK and Experimental Cancer Medicine Centres, University of Southampton, Southampton General Hospital, Southampton, SO16 6YD UK; 3 School of Pharmacy, University of East Anglia, Norwich, NR4 7TJ UK

**Keywords:** Androgen receptor, LSD1, Prostate cancer, Castration resistance, Enzalutamide, AR-V7 splice variant

## Abstract

**Background:**

Castrate resistant prostate cancer (CRPC) is often driven by constitutively active forms of the androgen receptor such as the V7 splice variant (AR-V7) and commonly becomes resistant to established hormonal therapy strategies such as enzalutamide as a result. The lysine demethylase LSD1 is a co-activator of the wild type androgen receptor and a potential therapeutic target in hormone sensitive prostate cancer. We evaluated whether LSD1 could also be therapeutically targeted in CRPC models driven by AR-V7.

**Methods:**

We utilised cell line models of castrate resistant prostate cancer through over expression of AR-V7 to test the impact of chemical LSD1 inhibition on AR activation. We validated findings through depletion of LSD1 expression and in prostate cancer cell lines that express AR-V7.

**Results:**

Chemical inhibition of LSD1 resulted in reduced activation of the androgen receptor through both the wild type and its AR-V7 splice variant forms. This was confirmed and validated in luciferase reporter assays, in LNCaP and 22Rv1 prostate cancer cell lines and in LSD1 depletion experiments.

**Conclusion:**

LSD1 contributes to activation of both the wild type and V7 splice variant forms of the androgen receptor and can be therapeutically targeted in models of CRPC. Further development of this approach is warranted.

**Electronic supplementary material:**

The online version of this article (10.1186/s12935-018-0568-1) contains supplementary material, which is available to authorized users.

## Background

Over 40,000 men are diagnosed with prostate cancer, and over 10,000 die from advanced metastatic disease, annually in the UK. Initial treatment of metastatic disease is reliant on inhibiting androgen receptor (AR) signalling by systemic androgen deprivation therapy (ADT). Although almost all patients respond to ADT, disease progression, to a clinical phenotype termed castration resistant prostate cancer (CRPC), occurs in virtually all patients. Metastatic CRPC is almost uniformly lethal within a median of 2–3 years and is commonly accompanied by significant symptomatic and healthcare burden [1].

Treatment options after transition to CRPC have developed significantly in the last few years. Approaches that are established to extend survival include further hormonal intervention with either the new generation AR antagonist enzalutamide or the CYP17A1 androgen synthesis inhibitor abiraterone acetate [2–5]. These therapeutic advances reflect the fact that, despite resistance to systemic androgen suppression, CRPC usually remains critically dependent on AR signalling.

As a conventional nuclear steroid hormone receptor transcription factor, AR activation involves ligand binding (e.g., testosterone, dihydro-testosterone), phosphorylation and homo-dimerisation, nuclear translocation, formation of co-regulator complex interactions with chromatin modifiers, androgen response element (ARE) sequence binding within target gene promoters and AR target gene expression and suppression. Non-genomic/non-ARE mediated effects also occur through AR activation [6].

Androgen deprivation therapy failure and transition to CRPC occurs through multiple mechanisms including expression of constitutively active AR point mutants and splice variants, AR co-regulator expression changes, AR expression change, altered AR ligand sensitivity/promiscuity and signalling pathway crosstalk (e.g., with PI3K/AKT or IGFR). Critically we have no current routinely available means within the clinic to detect resistance mechanisms to either initial ADT or subsequent therapies [6–12].

Intense interest currently surrounds the V7 splice variant of the AR which lacks a ligand (or enzalutamide) binding domain leaving a constitutively active N-terminal. AR-V7 expression represents a relatively common cause of transition to a CRPC phenotype and a cause of resistance to enzalutamide or abiraterone. Recent data found that AR-V7 detection in circulating tumour cells was possible in 19–39% of CRPC patients who were naive to new generation hormonal agents. AR-V7 expression was associated with almost complete loss of biochemical prostate specific antigen (PSA) response to enzalutamide or abiraterone and reduced median survival [7, 8]. Subsequent data have emerged to indicate that AR-V7 forms part of a wider spectrum of activating AR structural derangements, typically splice variants and point mutants, that drive some CRPC and with the hope that detection might allow for treatment resistance/sensitivity biomarkers to be developed [13].

Co-regulators of AR transcriptional activation include epigenetic mediators that induce chromatin remodelling at AR response element sites [14, 15]. A key example is the flavin-dependent nuclear amine oxidase LSD1 [16]. LSD1 is a lysine demethylase which, dependant on context, can repress or activate transcription. The best characterised LSD1 substrates for demethylation are mono- and di-methylation at lysine 4 of histone H3 (H3K4me1, H3K4me2) which are transcriptionally activating. LSD1 is a component of the CoREST transcriptional co-repressor complex that also contains CtBP and histone deacetylase (HDAC) 1 and HDAC2. Within this complex, LSD1 demethylates H3K4me1 and H3K4me2, facilitating gene silencing in concert with HDAC activity [16, 17]. By contrast, LSD1 associates with the AR in human prostate cells to remove mono- and di-methyl marks at histone H3 lysine 9 (H3K9me1, H3K9me2) at ARE sites [14, 15]. In contrast to H3K4 methylation, H3K9 methylation is transcriptionally repressive and the AR co-regulator function of LSD1 facilitates AR dependent transcriptional activation [14, 15]. LSD1 functions in concert with the histone demethylase lysine (K)-specific demethylase 4C, which demethylates H3K9me3 within AR activating complexes to promote AR mediated transcription also [14, 15]. Cellular epigenetic status in favour of H3K9 demethylation by LSD1 over H3K4 demethylation is not fully elucidated but determined in part through protein kinase C beta I phosphorylation of histone H3 threonine 6 [18]. Alternative splicing of LSD1 may also impact on substrate specificities in some contexts [19].

LSD1 expression is increased in prostate cancer compared with benign prostate and its expression correlates with higher Gleason score, risk of relapse and distant metastases, and reduced survival post prostatectomy [20, 21]. Histone marks consistent with LSD1 activity are altered in prostate cancer versus normal tissue (reduced H3K4me1, H3K9me2 and H3K9me3 marks) [22]. Taken together these data provide a rationale to develop LSD1 inhibition as a strategy to attenuate AR signalling.

We and others have shown that chemical inhibition of LSD1 inhibits cell proliferation and AR signalling in prostate cancer cells [14, 15, 23–25]. Validation of LSD1 as a potential therapeutic target in prostate cancer has been undertaken through LSD1 siRNA depletion experiments [14]. We hypothesised that LSD1 inhibition would also attenuate AR activation in models of CRPC driven by the AR-V7 splice variant.

## Methods

### Reagents and cell lines

Cell lines were maintained in Dulbecco’s Modified Eagle’s Medium (HEK293 cells; Sigma) or Roswell Park Memorial Institute (RPMI) medium (LNCaP cells; Sigma) supplemented with 10% fetal bovine serum (FBS) with 4 mM (HEK293) or 2 mM (LNCaP) l-glutamine and 1 mM pyruvate. For experiments, LNCaP cells were incubated for 24 h and then media was changed to RPMI-1640 (phenol free; Life Technologies) supplemented, for the remainder of the experiment, with 10% charcoal stripped FBS (Life Technologies). Dihydrotestosterone (DHT) and 5α-Androstan-17β-*ol*-3-one were from Sigma-Aldrich; LSD1 inhibitors LSD1-C76 and HCI-2509 were from Xcess Biosciences; enzalutamide and apalutamide were from Selleckchem. Chemical inhibitors of LSD1 were synthesised, and a separate publication will describe full details of the preparation.

### In vitro LSD1 inhibition assay

IC_50_ values for LSD1 activity after chemical inhibition were measured as previously described [25]. Briefly, this involves incubation of the purified enzyme with an H3K4me2 peptide substrate and measurement of by-product H_2_O_2_ using AmplexRed^®^ (Invitrogen).

### Cell proliferation assays

Cell proliferation assays were performed by incubating cells in 96 well plates in RPMI complete medium. After 24 h, different drugs or a DMSO control were added and analysis of proliferation was determined after 72 h using the CellTiter-Glo^®^ 2.0 Assay (Promega) according to the manufacturer instructions. Luminescence was measured using a Varioskan Flash Multimode Reader (Thermo Scientific).

### Immunoblotting

Cells were harvested and lysed by resuspension in protein sample buffer. DNA from samples was fragmented by sonication. Total protein from each sample was separated using the Laemmli method [26]. After blocking, the membranes were immunoblotted overnight at 4 °C with primary antibodies to LSD1, PSA/KLK3, androgen receptor and cleaved PARP (Cell Signalling) and β-Actin (Sigma-Aldrich) as previously described [27].

### CRISPR experiment

LSD1 knockout was undertaken in HEK293 cells according to the protocol from Zhang et al. [28]. Briefly, HEK293 cells were transfected with the Cas9 expressing vector pSpCas9(BB)-2A-GFP (Addgene) with a guide sequence targeting the ATG for *KDM1A* (*LSD1*). For the selection of the guide sequence oligonucleotide we used an online tool for CRISPR design (http://crispr.mit.edu/). Guide sequence oligonucleotides were, top 5′ caccgTGTGTTTTGATCGGGTGTTC 3′ and bottom 5′ aaacGAACACCCGATCAAAACACAc 3′.

### Luciferase reporter assays

Luciferase assays were performed using a vector (pARE-Luc) with the firefly luciferase gene under the control of the androgen receptor response element (ARE) in HEK293 cells co-transfected with peAR-Wt and peAR-V7 plasmids and with a renilla luciferase vector used as a transfection efficiency control as previously described [29, 30]. Briefly, HEK293 cells were transfected with Fugene HD (Promega) using a 9:1 ratio of AR:ARE vectors (1 µg DNA) along with 5 ng of a renilla control vector. After 30 h, cells were treated with chemical inhibitors according to the experimental requirements and then firefly and renilla luciferase activity were measured using the Dual-Luciferase^®^ Reporter Assay System (Promega) in a Varioskan™ Flash Multimode Reader (Thermo Scientific). All results show firefly luciferase activity normalised to renilla luciferase activity to control for transfection efficiency.

### Chromatin immunoprecipitation (CHIP) assay

CHIP assays were performed using the ChIP-IT^®^ High Sensitivity kit (Active Motif) according to the manufacturer’s instructions. Immunoprecipitation of the chromatin bound DNA was performed using the following antibodies from Active Motif: Histone H3 K9me3, Histone H3, Histone H3 K9me2, Histone H3K9me1. The ChIP-IT^®^ Control Kit, Human was used with these assays as a control.

### Quantitative real-time PCR

Total mRNA extraction was performed using the RNAeasy kit (Qiagen) according to the manufacturer’s instructions. Contaminating DNA was digested using RQ1 DNAse (Promega). Synthesis of cDNA was performed using M-MLV Reverse Transcriptase (Promega) according to the manufacturer’s instructions. For qRT-PCR we used the TaqMan^®^ Universal PCR Master Mix, No AmpErase^®^ UNG (ThermoFischer Scientific) and TaqMan^®^ Gene Expression Assays probes for KLK3 (PSA) and for the reference gene GAPDH. Assays were performed in an Applied Biosystems 7500 Real Time PCR System. Quantification of mRNA expression levels was performed using the 2^−∆∆C(T)^ method as described [31].

## Results

### Chemical inhibition of LSD1 inhibits activity of the androgen receptor

**Fig. 1:**
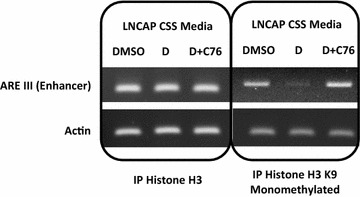
CHIP analysis of LNCaP cells treated with the LSD1 inhibitor LSD1-C76. LNCaP cells were incubated in media with charcoal stripped serum (CSS) for 24 h prior to addition of dihydrotestosterone (D, 1 nM) ± LSD1-C76 (C76, 250 μM) for 24 h. CHIP was performed using histone H3 and histone H3 K9 mono-methylated antibodies. Precipitated chromatin was amplified using primers flanking the enhancer region (ARE III) of the PSA gene or actin gene promoter [14]. *DMSO* dimethyl sulfoxide solvent control

**Fig. 2:**
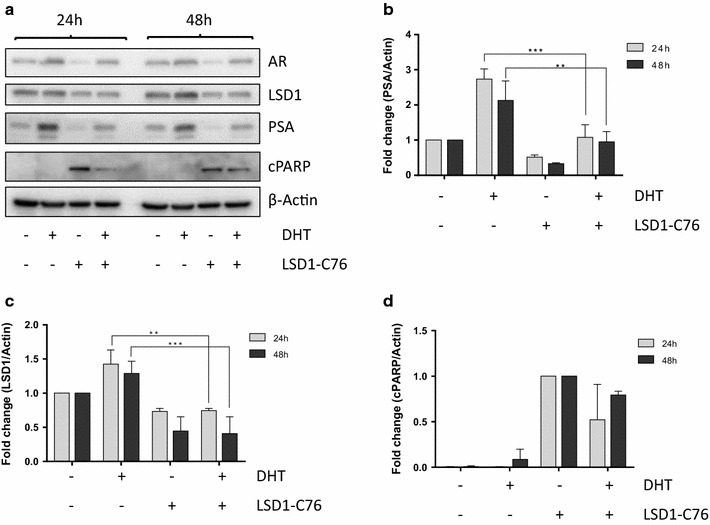
Chemical inhibition of LSD1 reduces AR, PSA and LSD1 expression and induces PARP cleavage. **a** Western blot analysis of the indicated targets in LNCaP prostate cancer cells, grown in charcoal stripped media for 24 h before exposure to dihydrotestosterone (DHT, 1 nM) or LSD1-C76 (250 μM) for 24 or 48 h as indicated. **b**–**e** Quantification of PSA (**b**), LSD1 (**c**), cPARP (**d**) and AR-WT (**e**) protein levels by densitometry analysis and normalized to β-actin. Western blots are representative of two independent experiments. P values were derived using 2 Way ANOVA multiple comparisons test. cPARP, cleaved PARP; ** and ***P < 0.005 and P < 0.001 respectively

We first validated the impact of LSD1 chemical inhibition on wild type androgen receptor transcriptional activation. We investigated the effects of the LSD1 inhibitor tranylcypromine (**1**, Additional file 1: Figure S1) which is currently under investigation in clinical trials for acute myeloid leukaemia (http://www.clinicaltrials.gov) and a set of five second-generation tranylcypromine analogues (**2**–**6**, Additional file 1: Figure S1) with improved target affinity in biochemical enzyme assays. Each of these compounds were shown to inhibit both in vitro activity of LSD1 and cell proliferation in LNCaP prostate cancer cells (Table 1, Additional file 1: Figure S2, Figure S3) but with many fold increase in potency over the tranylcypromine base compound. LSD1 chemical inhibition reversed DHT induced loss of histone H3 K9 mono-methylation in LNCaP prostate cancer cells in CHIP assays (Fig. 1). We also demonstrated reduction of AR and LSD1 protein expression and of the AR transcriptional target PSA in LNCaP cells, together with evidence of PARP cleavage in response to LSD1 inhibition (Fig. 2).

**Table 1:** IC_50_ and LD_50_ values for LSD1 inhibitor compounds for in vitro LSD1 inhibition assays and LNCaP prostate cancer cell proliferation assays respectively

Compound	LSD1 inhibition		Cell proliferation	
	IC_50_ mean (nM)	SD (nM)	LD_50_ mean (μM)	SD (μM)
Tranylcypromine (**1**)	57,980.0	5922.9	2235.0	173.6
2	4389.0	157.9	136.3	1.7
3	1520.5	890.3	605.9	90.4
4	259.3	22.8	224.9	27.9
5	269.4	35.7	712.4	48.5
6	221.6	26.7	183.9	19.6
LSD1-C76	527.0	9.0	85.1	9.5

Data represent mean values ± standard deviation (SD), as indicated, for a minimum of three experiments in each case

### LSD1 chemical inhibition attenuates AR-V7 splice variant activation

**Fig. 3:**
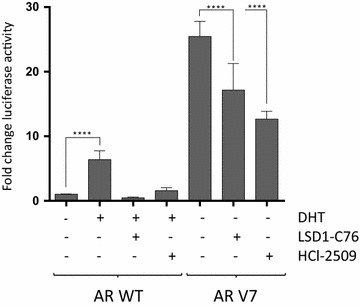
LSD1 chemical inhibition depletes AR response element activation through the AR-V7 splice variant. Luciferase reporter assay of AR response element (ARE) promotor activation in HEK293 cells co-transfected, 24 h after seeding as indicated, with either AR-WT or AR-V7 expression vectors, and incubated 24 h later as indicated with dihydrotestosterone (DHT, 1 nM), LSD1-C76 (250 μM) or HCl-2509 (5 μM) or DMSO solvent control and then analysed at 18 h. Samples were normalized against the AR-WT DMSO treated samples. Data are mean values ± standard deviation from 4 separate experiments each with triplicate determinations. P values were derived using a two-way ANOVA for comparison followed by Tukey’s multiple comparison post hoc tests. *AR* androgen receptor; *WT* wild type; ****P < 0.0001

AR-V7 and other AR splice variant forms are strongly implicated in hormonal therapy resistance and may contribute to some forms of castration resistant prostate cancer [7, 8]. To explore the impact of LSD1 on AR-V7 activity, we initially utilised a luciferase reporter assay system to measure ARE activation in HEK-293 cells through co-transfection of the AR with either wild type (WT) or V7 splice variant forms. Within this model system, we first confirmed that AR-WT activation is inhibited by the established AR antagonists enzalutamide [2, 5] and apalutamide [32, 33] but that the AR-V7 splice variant, by virtue of constitutive activation and loss of the binding site for either drug, demonstrates resistance to these agents (Additional file 1: Figure S4). Having confirmed the anticipated functional characteristics of this system we then demonstrated that partial inhibition of AR-V7 constitutive activation is seen upon chemical inhibition of LSD1 (Fig. 3).

### LSD1 depletion attenuates both AR-WT and AR splice variant activation

**Fig. 4:**
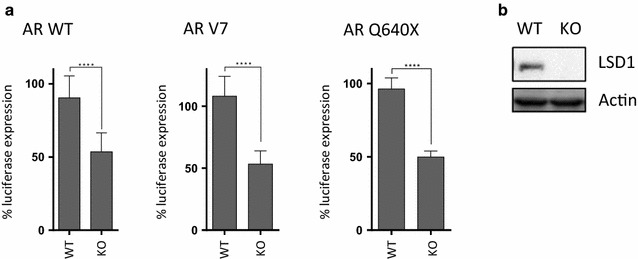
AR-V7 splice variant activation is attenuated by LSD1 depletion. **a** Luciferase androgen response element (ARE) reporter assays in HEK293 cells with (KO) or without (C) stable depletion of LSD1 (by CRISPR) followed by transient co-transfection with an ARE luciferase reporter construct and either AR-WT, AR-V7 or AR Q640X expression vectors as indicated. In the cells transfected with AR-WT, dihydrotestosterone (1 nM) was added for 18 h. Samples were normalized against LSD1 WT DMSO treated samples for the respective AR-WT of AR variant form transfected. Data are mean ± SD (n = 3). P values were derived using a two-way ANOVA for comparison followed by Tukey’s multiple comparison post hoc tests. (Controls without transfection of either the AR or an AR variant produced no ARE luciferase signal in these experiments). **b** Western blot of HEK293 cell LSD1 expression with (KO) or without (WT) stable depletion of LSD1. ****P < 0.0001

To validate evidence that LSD1 contributes to the activity of AR splice variant forms we undertook experiments to deplete LSD1 expression in HEK-293 cells followed by co-transfection of either AR-WT or splice variant forms in ARE luciferase reporter experiments. Depletion of LSD1 (by CRISPR; Additional file 1: Figure S5) resulted in partial inhibition of AR activity in this model for both the AR-WT and the AR-V7 splice variant and also for a further, constitutively active, AR C-terminal truncated splice variant Q640X (Fig. 4) [29].

**Fig. 5:**
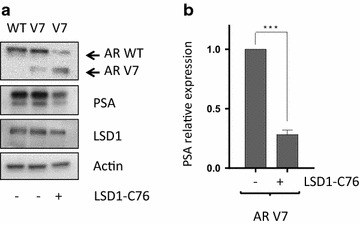
Chemical inhibition of LSD1 reduces PSA expression driven by AR-V7. **a** Western blot analysis of AR, LSD1 and PSA expression in LNCaP cells transfected with either the AR-WT or the AR-V7 splice variant as indicated. LNCaP cells were incubated in CSS-media for 24 h prior to transfection and after 24 h LSD1-C76 (250 μM) or DMSO solvent control were added for 18 h. **b** Quantification of PSA expression in three independent experiments. PSA levels were normalized against the DMSO treated samples. P values were derived using an unpaired t test with Welch’s correction. ***P < 0.05

We next turned to a prostate cancer cell line model for AR-V7 activation through transfection of an AR-WT or AR-V7 expression construct into the LNCaP cell line. In this model, chemical inhibition of LSD1 resulted in depletion of AR-WT and LSD1, although not AR-V7 in this model, and also the AR transcriptional target PSA (Fig. 5).

### LSD1 inhibition depletes AR activity in prostate cancer cells expression endogenous AR-V7

**Fig. 6:**
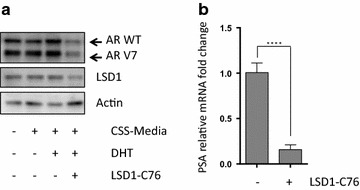
Chemical inhibition of LSD1 depletes AR activity in an AR-V7 expressing 22Rv1 prostate cancer cells. 22Rv1 cells, expressing both endogenous AR-WT and V7 splice variants, were incubated in CSS-media for 24 h prior to adding dihydrotestosterone (DHT, 1 nM) and LSD1-C76 (250 μM) for 18 h as indicated (or DMSO solvent control). **a** Western blot analysis of AR and LSD1 expression. **b** PSA mRNA expression determined by qPCR in three independent experiments. P values were derived using an unpaired t test with Welch’s correction. ****P < 0.05

Finally we utilised the 22Rv1 prostate cancer cell line to represent a CRPC model with high AR activity and endogenous expression of both the AR full length and V7 forms [34]. In 22Rv1 cells we showed reduced expression of both AR-WT, AR-V7 and LSD1 and also depletion of PSA RNA levels on exposure to chemical inhibition of LSD1 (Fig. 6).

## Discussion

Castration resistant prostate cancer is a challenging disease. Although there have been significant advances in new therapeutic options, including hormonal therapy, chemotherapy, systemic radioisotope therapy and immunotherapy, the disease remains highly lethal [2–5, 33, 35–37]. In fact, CRPC normally remains critically dependent on AR signalling, even following acquired resistance to hormonal interventions such as enzalutamide, apalutamide and abiraterone [7, 8]. The mechanisms underlying castration resistance commonly relate to the AR itself through receptor amplification, activating point mutations and constitutively active truncating splice variants of which the AR-V7 splice variant is a key example. One strategy to develop novel approaches to CRPC is therefore to target abnormal forms of the AR, including constitutively active splice variants, of which AR-V7 is the most frequently relevant form [7, 8].

Epigenetic therapeutic approaches remain experimental in prostate cancer. However, evidence exists for a variety of epigenetic mechanisms driving prostate cancer development, disease progression and aggressiveness, and transition to a CRPC phenotype. Relevant epigenetic aberrations include silencing of tumour suppressor genes by promoter hypermethylation, aberrant expression of histone modulating proteins, and DNA hypomethylation [38]. Potential approaches to target these are therefore of interest. DNA methyltransferase inhibition and histone deacetylase inhibition have received the most attention although translation through to clinical benefit remains to be established, perhaps partly due to a lack of companion predictive biomarkers for these approaches [38]. LSD1 has been shown to attenuate wild type AR activity and drive progression of the disease to more advanced clinical states [15, 20–24, 38]. We therefore sought to test whether this would extend to an impact on the function of AR-V7 as a key driver of many cases of CRPC and hormonal therapy resistance [7, 8].

We have established, in a variety of models, that LSD1 inhibition will attenuate signalling through not just the WT form of the receptor but also the ligand independent constitutively active AR-V7 form. Evidence for our ability to target the receptor with chemical inhibition was validated through use of knockdown experiments to deplete LSD1 expression. In addition we have demonstrated this in both a HEK293 co-transfection model to facilitate isolation of this phenomenon to AR-V7 or AR-WT separately, as well as in prostate cancer cell line models for dual V7 and WT expression through either forced expression (LNCaP cells) or endogenous expression (22Rv1 cells). We therefore propose that the impact of LSD1 on the androgen receptor is likely not lost by transition to a castration resistant phenotype where this arises through AR-V7 activity and regardless of the WT/V7 co-expression status. This would imply that AR-V7 retains a requirement for complex formation with LSD1, for functional activation. Having established that AR-V7 driven prostate cancer remains susceptible to targeting of LSD1, at least in the model systems used here, it will be important to dissect the molecular interaction between chemical LSD1 inhibition and an LSD1:AR interaction. Proper understanding of the molecular basis for a therapeutic effect will be critical to facilitate exploitation of this strategy. In addition, we fully acknowledge that LSD1 inhibition might function as a therapeutic strategy through other mechanisms beyond targeting of AR-WT and AR-V7.

Our results would suggest broadly that depletion of LSD1 activity was able to reduce AR-V7 activation by about 50% in most of the experimental models that we utilised. This was seen in LSD1 knockdown models and so implies that this may be more than a simple ‘drug potency’ issue. It is likely that other epigenetic influences on AR activation remain relevant and thus combinatorial approaches may be required for optimal suppression of AR function [38]. To what degree a greater impact on AR signalling, either through the wild type or V7 splice variant forms of the AR will be required for therapeutic efficacy remains to be determined. Our work has shown that reduced LSD1 activity is effective in attenuating signalling through the AR Q640X splice variant in addition to AR-V7 suggesting that this experimental approach might be considered more widely than just ‘AR-V7 positive’ CRPC. CRPC driven through other AR activating mechanisms, such as activating point mutations, AR amplification, AR phosphorylation or AR methylation should be investigated also.

## Conclusions

AR-V7 splice variant models of CRPC are amenable to therapeutic targeting through LSD1 inhibition. This raises the opportunity to develop this therapeutic strategy further.

## Additional file

**Additional file 1: FIgure S1.** Figure S1 Molecular structure of the LSD1 inhibitors used in this work. **Figure S2.** In vitro LSD1 inhibition assay for the indicated compounds. Data points show percentage of LSD1 activity when compared with the DMSO treated sample for three separate experiments. **Figure S3.** Cell proliferation assays for the indicated compounds in LnCAP prostate cancer cells. Data points show percentage luminescence compared to DMSO solvent exposed control samples after 72 h for a minimum of three separate experiments in each case. **Figure S4**. Androgen response element (ARE) luciferase reporter assay, in response to androgen receptor (AR) antagonists, for the AR wild type (WT) or V7 splice variant forms. (A) Luciferase reporter assay of ARE promotor activation in HEK293 cells co-transfected, as indicated, with either AR WT or V7 expression vectors, and incubated as indicated with dihydrotestosterone (DHT; 1nM), enzalutamide (10uM) or apalutamide (10uM). Data are mean values ± standard deviation from four replicate determinations. P values were derived using a two-way ANOVA for comparison followed by Tukey's multiple comparison post hoc tests. (B) Western blot to confirm AR expression following transfection of either the WT or V7 or Q640X splice variant forms or an untransfected sample, as indicated, in HEK293 cells. ****, P < 0.0001; ns, non-significant; NT, non-transfected. **Figure S5.** Western blot analysis of LSD1 expression for different clones obtained after the CRISPR experiment. (A) immunoblot for the N-terminus of human LSD1. (B) immunoblot for the C-terminus of human LSD1. Actin expression is shown as a protein loading control.

## Abbreviations

ADT: androgen deprivation therapy
AR: androgen receptor
ARE: androgen response element
CHIP: chromatin immunoprecipitation
CRPC: castration resistant prostate cancer
DHT: dihydrotestosterone
HDAC: histone deacetylase
H3K4me1, H3K4me2: mono- and di-methylation respectively at lysine 4 of histone H3
H3K9me1, H3K9me2: mono- and di-methylation respectively at histone H3 lysine 9
PSA: prostate specific antigen
RPMI: Roswell Park Memorial Institute
V7: V7 splice variant
WT: wild type
