# Supplementary material for: LSD1 inhibition attenuates androgen receptor V7 splice variant activation in castration resistant prostate cancer models
Source: Cancer Cell Int. 2018 May 9;18:71. doi: 10.1186/s12935-018-0568-1 (PMC5941811; doi:10.1186/s12935-018-0568-1)
Supplement: Supplementary file 1 — Additional file 1: FIgure S1. Figure S1 Molecular structure of the LSD1 inhibitors used in this work. Figure S2. In vitro LSD1 inhibition assay for the indicated compounds. Data points show percentage of LSD1 activity when compared with the DMSO treated sample for three separate experiments. Figure S3. Cell proliferation assays for the indicated compounds in LnCAP prostate cancer cells. Data points show percentage luminescence compared to DMSO solvent exposed control samples after 72 h for a minimum of three separate experiments in each case. Figure S4. Androgen response element (ARE) luciferase reporter assay, in response to androgen receptor (AR) antagonists, for the AR wild type (WT) or V7 splice variant forms. (A) Luciferase reporter assay of ARE promotor activation in HEK293 cells co-transfected, as indicated, with either AR WT or V7 expression vectors, and incubated as indicated with dihydrotestosterone (DHT; 1nM), enzalutamide (10uM) or apalutamide (10uM). Data are mean values ± standard deviation from four replicate determinations. P values were derived using a two-way ANOVA for comparison followed by Tukey's multiple comparison post hoc tests. (B) Western blot to confirm AR expression following transfection of either the WT or V7 or Q640X splice variant forms or an untransfected sample, as indicated, in HEK293 cells. ****, P < 0.0001; ns, non-significant; NT, non-transfected. Figure S5. Western blot analysis of LSD1 expression for different clones obtained after the CRISPR experiment. (A) immunoblot for the N-terminus of human LSD1. (B) immunoblot for the C-terminus of human LSD1. Actin expression is shown as a protein loading control. [file 12935_2018_568_MOESM1_ESM.pptx]

## Slide 1
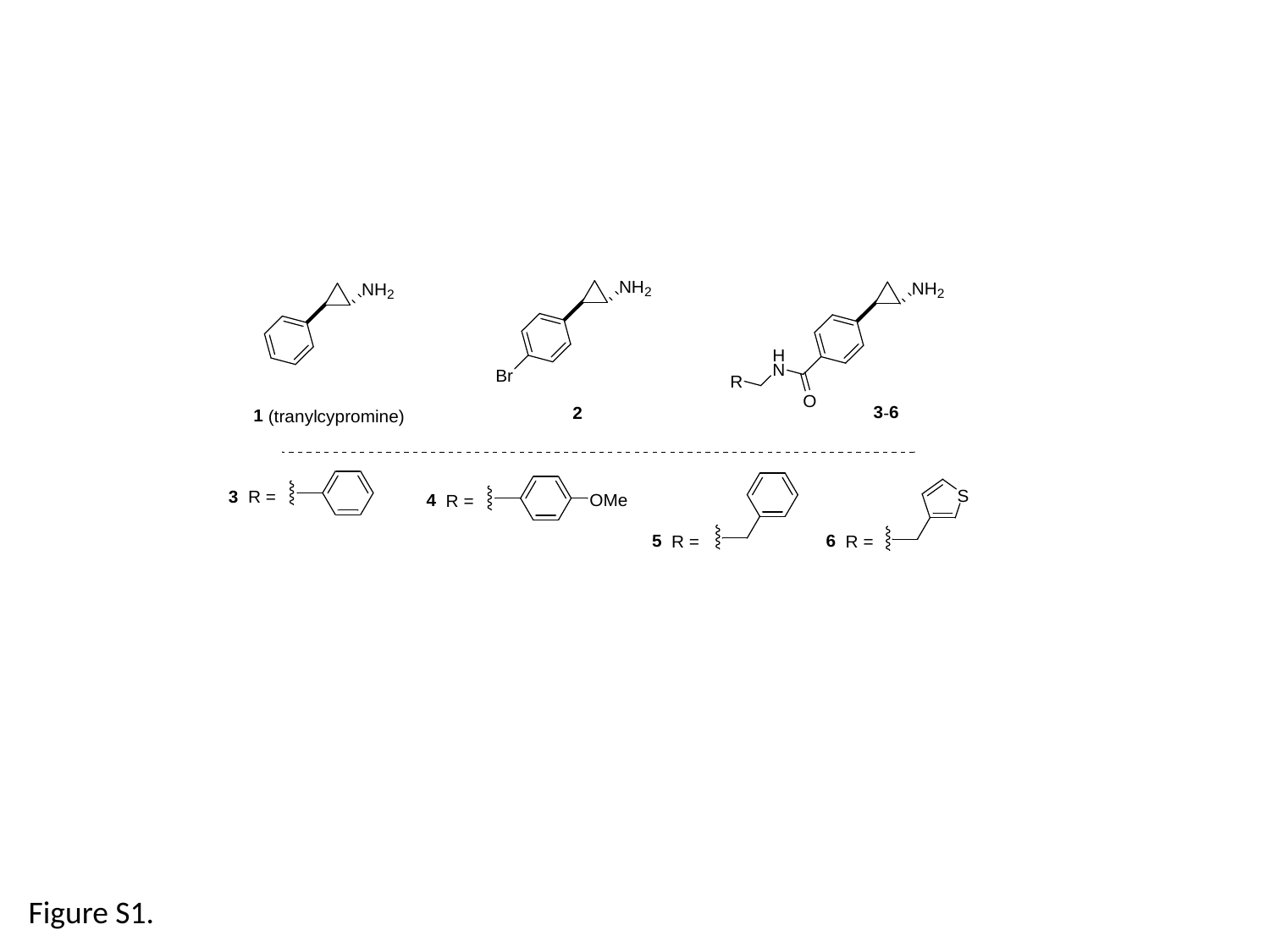

Figure S1.

## Slide 2
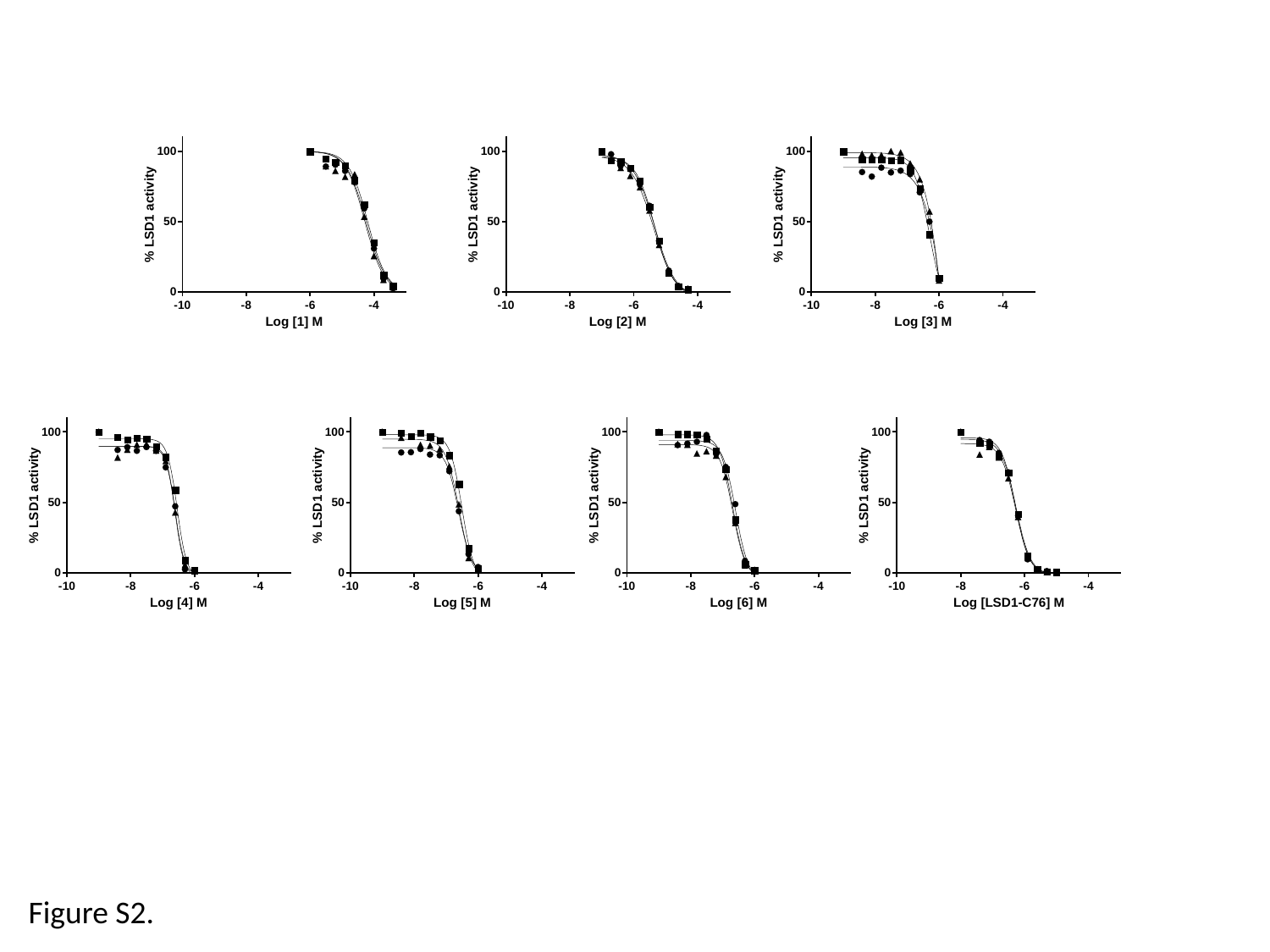

Figure S2.

## Slide 3
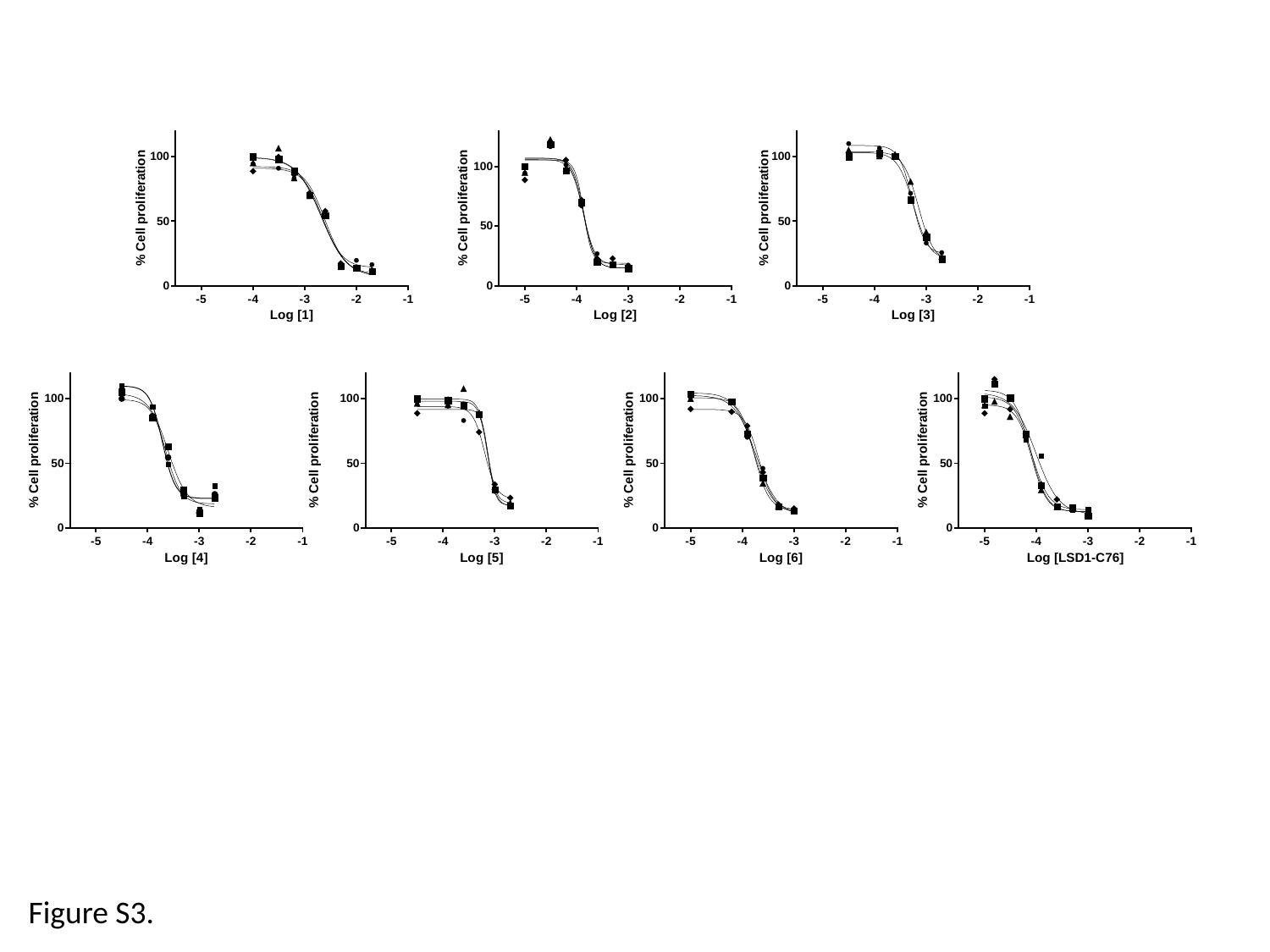

Figure S3.

## Slide 4
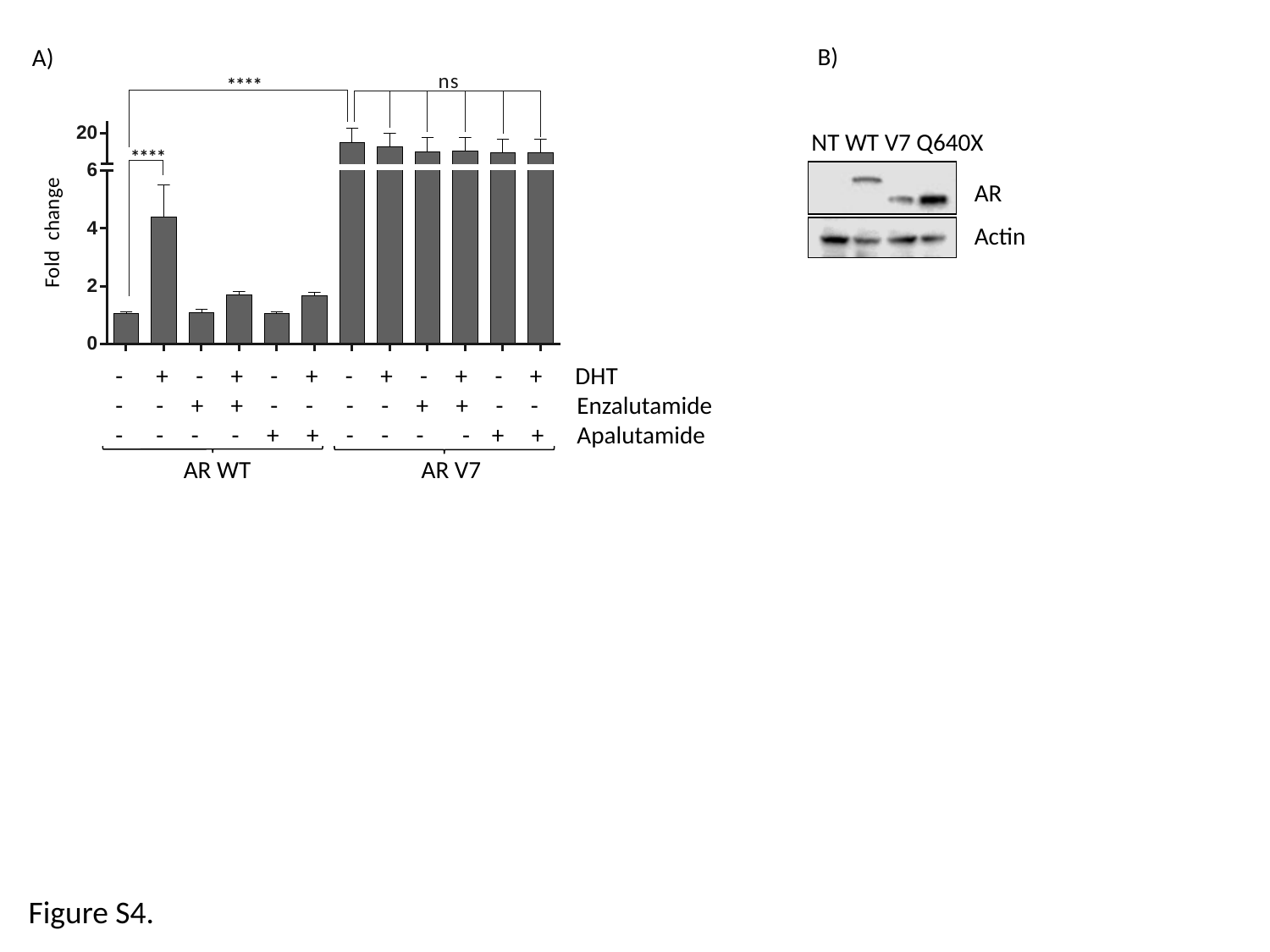

B)
A)
NT WT V7 Q640X
AR
Actin
- + - + - + - + - + - + DHT
- - + + - - - - + + - - Enzalutamide
- - - - + + - - - - + + Apalutamide
 AR WT AR V7
Figure S4.

## Slide 5
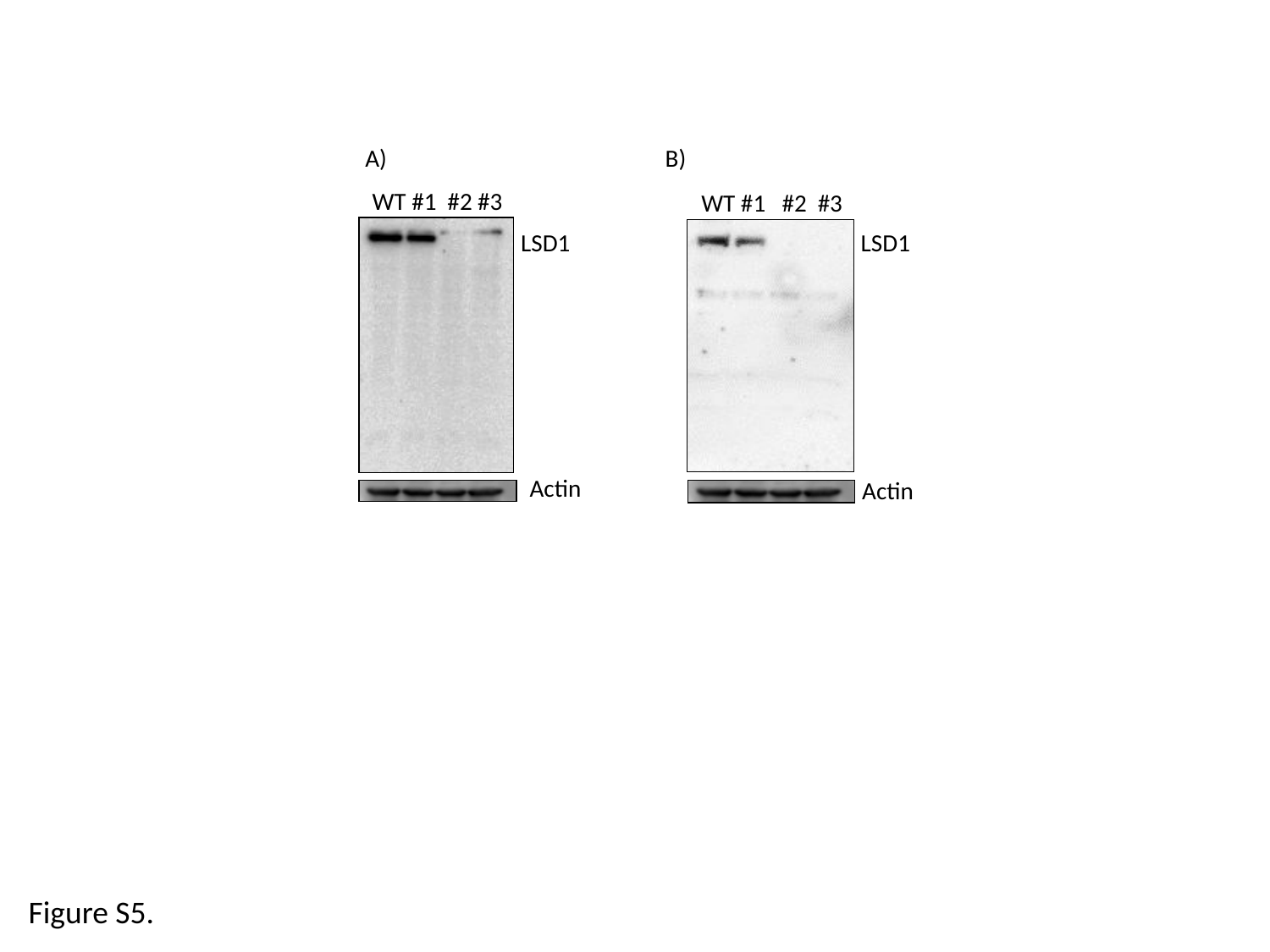

A)
B)
WT #1 #2 #3
LSD1
Actin
WT #1 #2 #3
LSD1
Actin
Figure S5.
